# Relationships between Chromosome 7 Gain, *MET* Gene Copy Number Increase and MET Protein Overexpression in Chinese Papillary Renal Cell Carcinoma Patients

**DOI:** 10.1371/journal.pone.0143468

**Published:** 2015-12-04

**Authors:** Xiaolu Yin, Tianwei Zhang, Xinying Su, Yan Ji, Peng Ye, Haihua Fu, Shuqiong Fan, Yanying Shen, Paul R. Gavine, Yi Gu

**Affiliations:** 1 Asia & Emerging Markets iMed, AstraZeneca R&D, Shanghai, China; 2 Research & Development Information, AstraZeneca R&D, Shanghai, China; 3 Department of Pathology, Ren Ji Hospital, School of Medicine, Shanghai Jiao Tong University, Shanghai, China; UCSF / VA Medical Center, UNITED STATES

## Abstract

To investigate the relationships between Chromosome 7 gain, *mesenchymal-epithelial transition factor* (*MET)* gene copy number increase and MET protein overexpression in Chinese patients with papillary renal cell carcinoma (PRCC), immunohistochemistry (IHC), immunofluorescence (IF) and fluorescence *in situ* hybridization (FISH) were performed on 98 formalin-fixed, paraffin-embedded (FFPE) PRCC samples. Correlations between *MET* gene copy number increase, Chromosome 7 gain and MET protein overexpression were analyzed statistically. A highly significant correlation was observed between the percentage of tumor cells with *MET* gene copy number ≥3 and CEP7 copy number ≥3 (R^2^ = 0.90, *p*<0.001) across two subtypes of PRCC. In addition, the percentage of tumor cells with *MET* gene copy number ≥3 was found to increase along with increases in MET IHC score. This correlation was further confirmed in those PRCC tumor cells with average *MET* gene copy number >5 using combined IF and FISH methodology. Overall, this study provides evidence that Chromosome 7 gain drives *MET* gene copy number increase in PRCC tumors, and appears to subsequently lead to an increase in MET protein overexpression in these tumor cells. This supports MET activation as a potential therapeutic target in sporadic PRCC.

## Introduction

Papillary renal cell carcinoma (PRCC) is the second most common subtype of renal cell carcinoma (RCC) and accounts for 10% ~ 15% of all RCC in the West, with clear cell renal cell carcinoma (CRCC) accounting for 80% of all RCC [1, 2]. Previous studies by Delahunt and Eble have divided PRCC into two morphologically different subtypes [3]. Type 1 PRCC is characterized by papillae covered by cells with scanty cytoplasms arranged in a single layer on the papillary basement membrane, while Type 2 PRCC is characterized by cells with eosinphilic cytoplasms and pseudostratified nuclei on papillary cores. Besides the morphological differences, Type 2 PRCC is usually more aggressive and presents a higher nuclear grade than Type 1 PRCC [3]. Unlike CRCC, where targeted therapy against vascular endothelial growth factor (VEGF) has dramatically improved the outcome of patients [4], VEGF-targeted agents show poor efficacy in PRCC. Up to now, no specific systematic therapy is available for metastatic PRCC [1].

Mesenchymal-epithelial transition factor (MET) protein functions as a transmembrane tyrosine kinase receptor [5]. When bound to its only known ligand, hepatocyte growth factor (HGF), MET protein activates downstream signaling pathways which promote cell proliferation, migration, invasion, angiogenesis and prevent cells from apoptosing [5]. It has been shown that germline mutations in *MET* lead to the development of hereditary Type 1 PRCC [6–8], sparking interest in the development of MET inhibitors to treat PRCC patients. Savolitinib, a MET inhibitor, was reported to induce tumor regressions in PRCC patient-derived xenograft models [9], and a phase II clinical trial to evaluate its efficacy in PRCC patients was recently launched (ClinicalTrials.gov. Maryland: the U.S. National Institutes of Health, Inc.; NCT02127710 [updated 2015 May 17]. Available from: https://clinicaltrials.gov/ct2/show/NCT02127710. Accessed May 26, 2015.). In sporadic PRCC patients, *MET* gene mutation [7], gene copy number alteration [10] and MET protein overexpression [10–13] were also observed. Recently, a study by Albiges *et al* reported *MET* gene copy number increases accompanied with high MET mRNA expression in a large cohort of 220 French PRCC patients [10]. Meanwhile, chromosome 7, where the *MET* gene resides, frequently exhibits trisomy in PRCC [14–21], also indicative of the occurrence of MET gene copy number increase in PRCC. Furthermore, tumors from PRCC patients carrying *MET* gene mutations commonly show trisomy 7 with non-random duplicated mutant *MET* genes and one wildtype *MET* gene [20].

Nevertheless, the etiology of sporadic PRCC is still largely unknown especially in Asian patients, possibly due to the lower prevalence of the disease in Asia [22]. Thus, our study aimed to investigate the association of Chromosome 7 gain, *MET* gene copy number variation and MET protein expression level in PRCC tumor tissues from a cohort of Chinese patients.

## Materials and Methods

### Patients

Tumor samples were collected from 98 PRCC patients who underwent surgery between 2010 and 2013 at Ren Ji Hospital, Shanghai, China. Prior written informed consent was obtained from all patients and the study protocol was approved by the ethics committee at Ren Ji hospital. Adjuvant chemotherapy was administered to 6 patients, while 46 patients did not receive chemotherapy. Chemotherapy status for the rest of the 65 patients was not available. Survival data was only available for 54 patients and therefore overall survival was not included in the data analysis due to the low follow-up response rate (55.1%). Histological subtypes (Type 1 and Type 2) were determined after review of tumor sections by two independent pathologists. This study was approved by Renji Hospital Institutional Review Board.

### Fluorescence in situ hybridization (FISH)

Dual-color FISH assay was performed as previously described [23] on tissue microarray (TMA) at 4 μm thickness. The *MET* probe was derived by labeling BAC (CTD-2270N20, Invitrogen) DNA with SpectrumRed-dUTP (Cat #02N34-050, Enzo Biochem). CEP7-SpectrumAqua probe, specifically targeting the centromere of chromosome 7, was purchased from Vysis (32–111007, Vysis) and used as an internal control. FISH signals were observed using a fluorescence microscope equipped with the appropriate filters to allow visualization of the intense red/aqua signals and the blue counterstained nuclei. *MET* gene and CEP7 copy number was scored on 100 tumor cells for each case. Cases with *MET* gene average copy number ≥6 were defined as *MET* amplification. The percentage of tumor cells with *MET* gene copy number increase (≥3) (%*MET*) and CEP7 copy number increase (≥3) (%CEP7) was further calculated to statistically assess the correlation between *MET* and chromosome 7 copy number variation by Spearman’s correlation analysis.

### Immunohistochemistry (IHC)

IHC analysis was performed on a 4 μm thick TMA. MET immunostaining was performed using CONFIRM anti-total c-MET (SP44) rabbit monoclonal antibody (790–4430, Ventana Medical Systems) on an automatic immunostainer (Discovery XT, Ventana Medical Systems) following standard staining protocol from Ventana Medical Systems. Ki67 IHC was performed as follows: slides were dried at 37°C overnight and then baked at 56°C for 30min, then deparaffinized in xylene and rehydrated through a graded series of ethanol concentrations. Antigen retrieval was performed in PTlink using high pH target retrieval solution at 97°C, 15 minutes (Target Retrieval Solution, pH 9, K8004, DAKO). Slides were covered with primary antibody solution (M7240, DAKO) and incubated at room temperature for 60 minutes, then washed twice in TBS-T and incubated with the EnVision+ system-HRP labelled polymer anti-rabbit secondary antibody (K4003, DAKO) for 30 minutes. Following two additional washes in TBS-T, slides were visualized using DAB substrate-chromagen (K3468, DAKO). All slides were independently evaluated and results were agreed by two pathologists who were blinded to patient information.

### Combined immunofluorescence (IF) and FISH assays

4 μm thick PRCC sections were firstly de-waxed and pretreated in high pH antigen retrieval solution (Tris-EDTA, pH = 9.0; K8004, DAKO) at 95°C for 30 minutes. Sections were then treated with protease 3 (760–2020, Ventana Medical Systems) for 6 minutes and then incubated in CONFIRM anti-total c-MET (SP44) rabbit monoclonal antibody for 1 hour. Goat anti-rabbit IgG FITC (5ug/ml, ab6717, Abcam) was added and sections were incubated for 30 minutes at room temperature. After post-IF fixation in neutrally-buffered formalin for 10 minutes, sections were dehydrated in ethanol series and air-dried. Probes for *MET* gene and CEP7 were applied and slides were co-denatured at 79°C for 6 minutes, and then incubated at 37°C for more than 30 hours. After overnight incubation, slides were washed with 0.3% NP40/2 x SSC (pH 7.0–7.5) at 72°C for 2 minutes, and then in 2 x SSC at room temperature for 2 minutes. Slides were finally dehydrated in ethanol series, air-dried, and mounted with mounting medium with DAPI (Cat #H-1200, Vector Labs). Slides were analyzed using fluorescent microscopy.

### Statistical analysis

Statistical analysis was performed using R statistical software, and *p*>0.05 was defined as statistically significant.

## Results

### Patient characteristics

**Fig 1:**
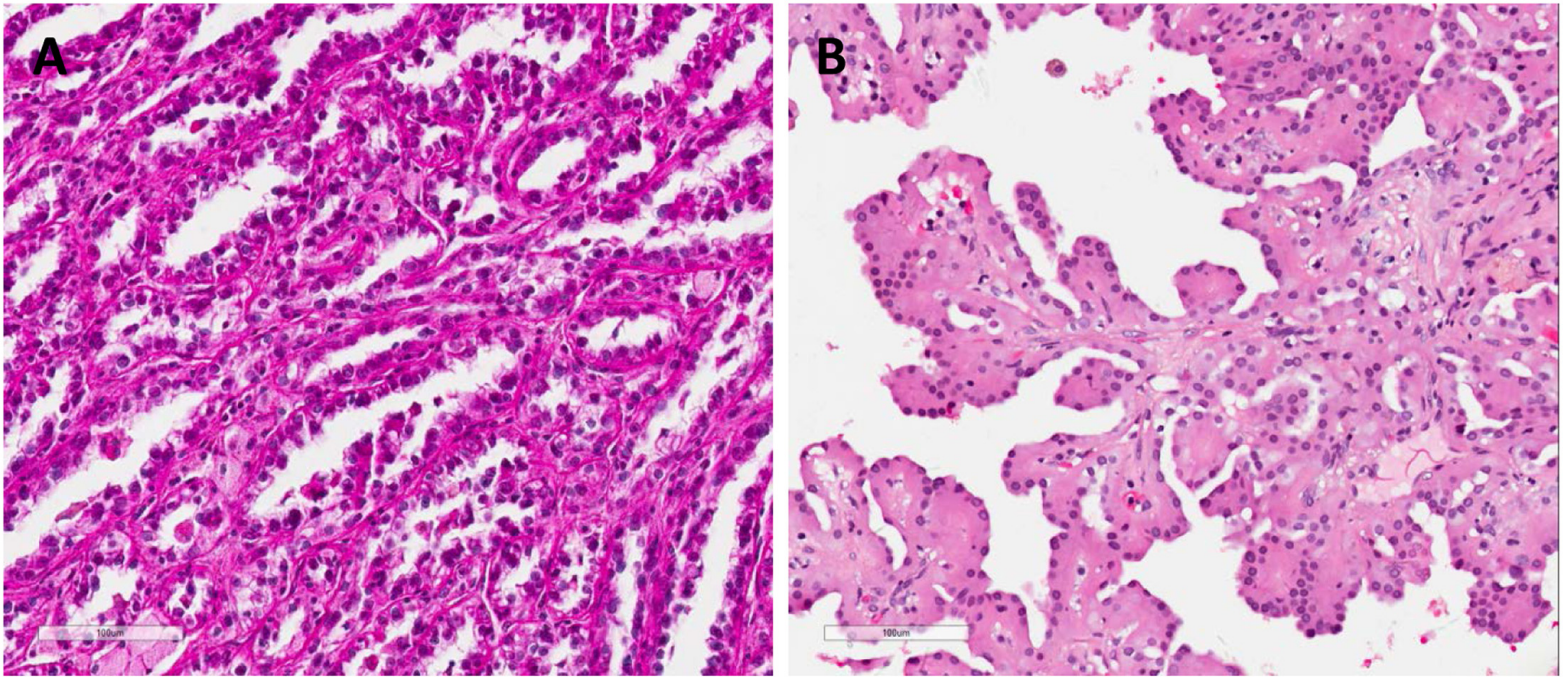
Representative images showing typical morphology in Type 1 (A) ad Type 2 (B) PRCC respectively. Type 1 PRCC was characterized by papillae covered by cells with scanty cytoplasms which were arranged in a single layer on the papillary basement membrane, while Type 2 PRCC was characterized by cells with eosinphilic cytoplasms and pseudostratified nuclei on papillary cores.

Detailed patient demographics and tumor characteristics are listed in Table 1. PRCC was more prevalent within males in this cohort (3:1), with the two subtypes almost evenly distributed (Type 1, 45.9% versus Type 2, 54.1%). The percentages of tumor grades were Grade 1, 8.2%; Grade 2, 48.0%; Grade 3, 32.6%; Grade 4, 11.2%. The percentages of clinical stages were Stage 1, 67.3%; Stage 2, 9.2%; Stage 3, 11.2%; Stage 4, 9.2%; Unknown 3.1%. Following independent review by two pathologists, all 98 samples were classified as PRCC (Fig 1). Consistent with previous studies [3], Type 2 PRCC was found to be correlated with higher tumor grades (*p*<0.001) and clinical stages (*p* = 0.01), when compared with Type 1 PRCC.

**Table 1:** 

Patient Demographics and Tumor Characteristics (N = 98). Factors	Number	%
**Age, years**		
≤ Median (58)	45	45.9
>Median (58)	45	45.9
Unknown	8	8.2
**Gender**		
Male	71	72.4
Female	27	27.6
**Histological type**		
Type 1	45	45.9
Type 2	53	54.1
**Tumor grade**		
Grade 1	8	8.2
Grade 2	47	48.0
Grade 3	32	32.6
Grade 4	11	11.2
**T**		
1a	47	48.0
1b	21	21.4
2	11	11.2
3	9	9.2
4	7	7.1
Unknown	3	3.1
**N**		
0	89	90.8
1	6	6.1
Unknown	3	3.1
**M**		
0	95	96.9
1	0	0
Unknown	3	3.1
**Clinical stage**		
1	66	67.3
2	9	9.2
3	11	11.2
4	9	9.2
Unknown	3	3.1

### Biomarker evaluation

For biomarker evaluation, samples which failed quality control (no tumor tissue found or failed assays) were excluded. MET protein expression was analyzed in 94 PRCC samples using IHC staining. The MET protein overexpression rate, as defined by IHC 2+ and 3+, was 73.4% (69/94). *MET* gene and chromosome 7 copy numbers were analyzed in 91 PRCC samples using FISH. Tumor cells with *MET* gene copy number ≥3 were observed in 90.1% (82/91) patients, while tumor cells with CEP7 copy number ≥3 were observed in 92.3% (84/91) patients. The percentage of tumor cells with *MET* or CEP7 copy number ≥3 was variable among patients. One case (1/91) of *MET* gene amplification (as defined by gene copy number ≥6), was observed in our cohort. Ki67 index, defined as the number of Ki67 positive cells per 1000 tumor cells, was evaluated in 98 PRCC samples. The overall Ki67 index was 0.65±1.72. Comparisons between Type 1 and Type 2 PRCC revealed similar biomarker prevalences (Table 2). All biomarkers showed no correlation with clinical parameters, with the exception that the Ki67 index showed a higher trend in male patients than females (*p*<0.001).

**Table 2:** Biomarker details in Type 1 and Type 2 PRCC.

PRCC subtype	MET protein expression (by IHC)		*MET* gene copy number (by FISH)		CEP7 copy number (by FISH)		Ki67 index (by IHC)
	low expression (IHC 0/1+)	high expression (IHC 2/3+)	disomy	with copy number ≥3	disomy	with copy number ≥3	Mean±SD
Type 1	16.3% (7/43)	83.7% (36/43)	7% (3/42)	93% (39/42)	5% (2/42)	95% (40/42)	0.66±1.53
Type 2	35.3% (18/51)	64.7% (33/51)	12% (6/49)	84% (43/49)	10% (5/49)	90% (44/49)	0.71±1.88
All	26.6% (25/94)	73.4% (69/94)	9.9% (9/91)	90.1% (82/91)	7.7% (7/91)	92.3% (84/91)	0.65±1.72

### Relationships between *MET* gene copy number increase and Chromosome 7 gain

**Fig 2:**
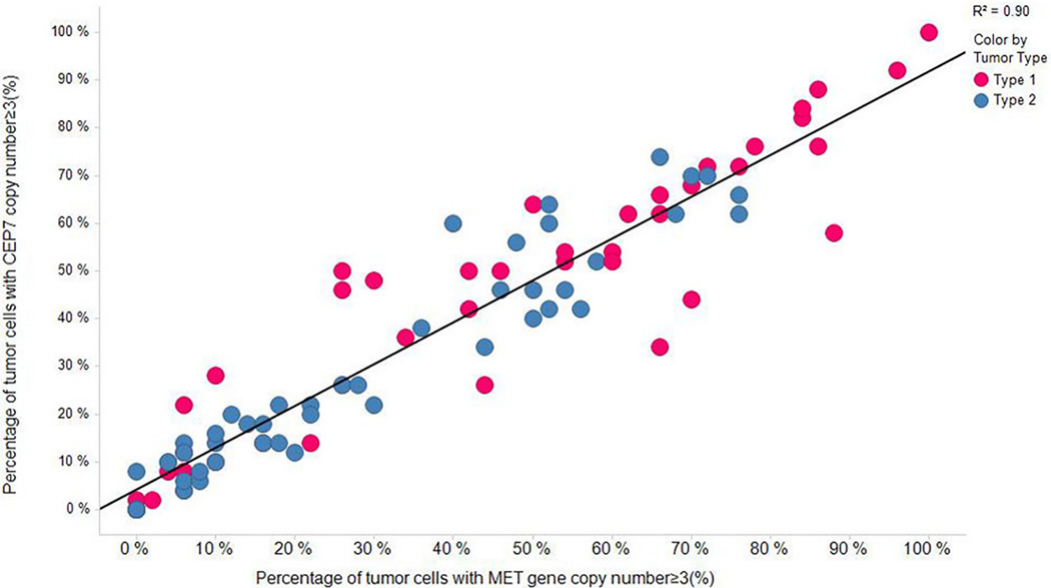
Correlation between percentage of tumor cells with *MET* gene copy number ≥3 and percentage of tumor cells with CEP7 gene copy number ≥3 in PRCC (N = 91) patients.

FISH assays were performed to determine the *MET* gene copy number and CEP7 copy number in 91 PRCC samples. After evaluation of *MET* and CEP7 copy numbers in the tumor cells, a significant correlation was found between the percentage of tumor cells with *MET* gene copy number increase (≥3) (%*MET*) and the percentage of tumor cells with CEP7 gene copy number increase (≥3) (%CEP7) (R^2^ = 0.90, *p*<0.001, Spearman’s correlation analysis, Fig 2). This indicates that copy number gains in Chromosome 7 are likely the cause of *MET* gene copy number increase in PRCC tumors. Such a correlation was observed in both PRCC tumor subtypes (R^2^ = 0.85 & 0.91, Type 1 and Type 2, respectively).

As shown in Fig 2, the %*MET* was significantly higher in Type 1 PRCC samples, as compared to Type 2 (*p* = 0.01). No significant relationship was found between %*MET* and clinical stage (*p* = 0.82), indicating that the increase in the percentage of tumor cells with *MET* gene copy number increase may not be directly related to the severity of the disease or tumor metastasis. After stratification of the tumor subtypes, no significant relationship was found between %*MET* and tumor grades (*p* = 0.89, Type 1; *p* = 0.73, Type 2) or clinical stages (*p* = 0.82, Type 1; *p* = 0.54, Type 2). Similar results were observed in %CEP7 (data not shown).

### Relationships between *MET* gene copy number increase and MET protein overexpression

**Fig 3:**
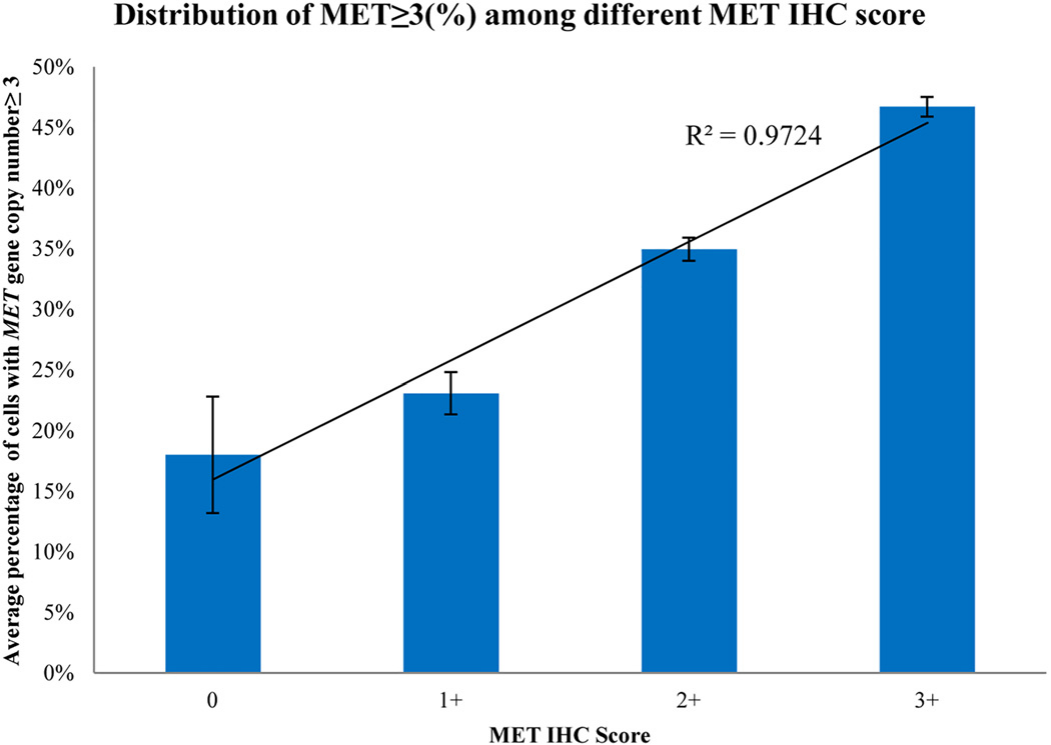
Distribution of percentage of tumor cells with *MET* gene copy number ≥3 among different MET IHC scores in PRCC (N = 88) patients.

**Fig 4:**
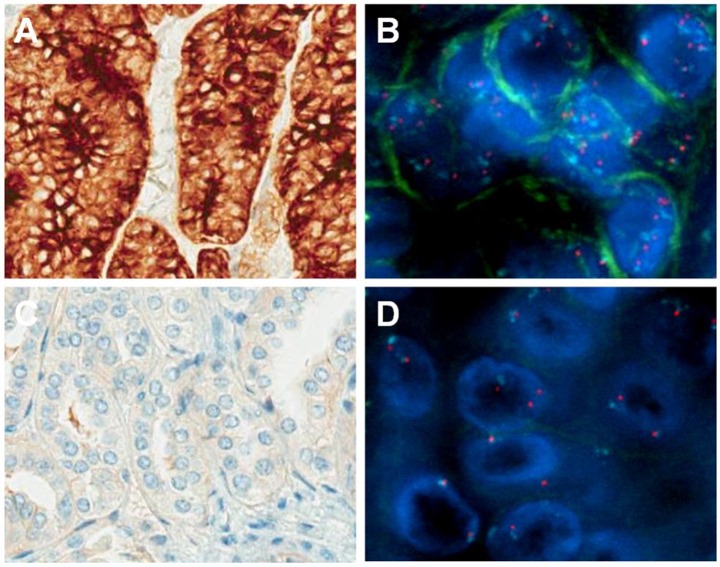
Correlation between MET protein overexpression and *MET* gene copy number increase. The upper row shows representative images of MET protein overexpression analyzed by both IHC (A) and IF (B) staining, correlating with *MET* gene copy number increase (average copy number >5) (B). The lower rows show representative images of no MET protein expression shown by both IHC (C) and IF (D) staining, correlating with *MET* disomy (average copy number = 2). For the fluorescent images, green signals represent IF staining which indicated by the triangle, red signals represent *MET* gene copy number which indicated by the arrows, aqua signals represent CEP7 copy number and nuclei are counterstained as blue.

A positive trend was observed between %*MET* and increasing IHC score (Fig 3), indicating a possible correlation between *MET* gene copy number increase and protein overexpression in PRCC tumor cells. Further studies using IF combined with FISH showed that MET protein overexpression (IF 3+) was colocalized in tumor tissues with average *MET* gene copy number >5 (PRCC-009, 052, 097; Fig 4). In contrast, in tumor tissue where MET IF showed negative staining, the average *MET* gene copy number = 2 (Fig 4). This result suggests a correlation between *MET* gene copy number increase and MET protein overexpression in the same tumor cells.

## Discussion

PRCC is the second most common type of renal carcinoma, and is associated with poor prognosis in some studies, especially in its metastatic form [24]. Hereditary Type 1 PRCC has been linked to a genetic mutation in the *MET* gene [6, 25]. In addition, in a previous phase II clinical trial, Foretinib (targeting VEGF/MET) showed a high (50%) response rate in PRCC patients with germline *MET* mutations [26]. However, the pathogenetic background and etiology of sporadic PRCC is still largely unknown. A recent study investigating 220 western patients with PRCC found a significant correlation between *MET* gene copy number and MET mRNA expression across the two subtypes of PRCC [10].

In this study, we used FISH and IHC techniques to investigate *MET* gene copy number variation and protein expression in 98 Chinese PRCC patients. Instead of calculating average gene copy number as previously reported, we looked into the detailed percentage of tumor cells with *MET* gene copy number ≥3 or with CEP7 gene copy number ≥3. In contrast to previous studies of whole tumor tissues using array CGH analysis, our detailed FISH scoring based on the percentage of tumor cells with *MET* gene copy number ≥3 should help to better evaluate gene copy number variations. Our results showed a highly significant correlation between %*MET* gene copy number increase (≥3) and %*CEP7* gene copy number increase (≥3) in PRCC tumor cells. Only a single case of *MET* gene amplification (≥6) was found in our PRCC sample set. These results suggest a role for Chromosome 7 trisomy or polysomy during the pathogenesis of sporadic PRCC. Chromosome 7 gain (trisomy 7) has also been observed in hereditary PRCC, and causes duplication of a mutated *MET* gene [20]. However, in other tumor types such as lung cancer and gastric cancer, *MET* gene amplification, without Chromosome 7 gain, was found to be a potent activator of *MET* pathway signaling [27–30], underscoring the complexity of MET pathway activation as an oncogenic driver across different tumor types.

Furthermore, we also found an increasing trend in the percentage of tumor cells with *MET* gene copy number ≥3 together with higher MET IHC score, suggestive of a relationship between *MET* gene copy number increase and MET protein overexpression. This confirms previous findings observed between *MET* gene copy number variation and MET mRNA expression [10]. Finally, our findings from the combined IF/FISH studies provide direct evidence that MET protein overexpression is observed in the same PRCC tumor cells with a high *MET* gene copy number increase (>5), but not in those tumor cells with normal *MET* gene copy number.

## Conclusions

Herein, we have studied the relationships between Chromosome 7 gain, *MET* gene variation and MET protein expression across the two subtypes of PRCC in a Chinese patient cohort. Our results clearly demonstrate that *MET* gene copy number increases in PRCC tumors are highly associated with increases in the copy number of Chromosome 7, which differs from previous observations in lung and gastric cancer. Such increases in *MET* gene copy number likely lead to increases in MET protein overexpression in PRCC tumor cells, especially in those with a high increase in the average *MET* gene copy number (>5). This study lends additional support to the concept of therapeutically targeting MET in sporadic PRCC patients.
